# High frequency of the recurrent c.1310_1313delAAGA *BRCA2* mutation in the North-East of Morocco and implication for hereditary breast–ovarian cancer prevention and control

**DOI:** 10.1186/s13104-017-2511-2

**Published:** 2017-06-02

**Authors:** Fatima-Zahra Laarabi, Ilham Ratbi, Siham Chafai Elalaoui, Loubna Mezzouar, Yassamine Doubaj, Laila Bouguenouch, Karim Ouldim, Noureddine Benjaafar, Abdelaziz Sefiani

**Affiliations:** 1 Centre de Génomique Humaine, Faculté de Médecine et Pharmacie, Université Mohammed V de Rabat, Rabat, Morocco; 2 Département de Génétique Médicale, Institut National d’Hygiène, 27, Avenue Ibn Batouta, B.P. 769, Rabat, Morocco; 3 Service de Radiothérapie, Centre d’Oncologie Hassan II, Oujda, Morocco; 4 Département de Génétique Médicale, Centre Hospitalier Universitaire Hassan II, Fès, Morocco; 5 Service de Radiothérapie, Institut National d’Oncologie, Rabat, Morocco

**Keywords:** Breast cancer, *BRCA2*, Recurrent mutation, North-East Morocco

## Abstract

**Background:**

To date, a limited number of *BRCA1/2* germline mutations have been reported in hereditary breast and/or ovarian cancer in the Moroccan population. Less than 20 different mutations of these two genes have been identified in Moroccan patients, and recently we reported a further *BRCA2* mutation (c.1310_1313delAAGA; p.Lys437IlefsX22) in three unrelated patients, all from the North-East of the country. We aimed in this study to evaluate the frequency and geographic distribution of this *BRCA2* frameshift mutation, in order to access its use as the first-line *BRCA* genetic testing strategy for Moroccan patients. We enrolled in this study 122 patients from different regions of Morocco, with suggestive inherited predisposition to breast and ovarian cancers. All subjects gave written informed consent to *BRCA1/2* genetic testing. According to available resources of our lab and enrolled families, 51 patients were analyzed by the conventional individual exon-by-exon Sanger sequencing, 23 patients were able to benefit from a BRCA next generation sequencing and a target screening for exon 10 of *BRCA2* gene was performed in 48 patients.

**Results:**

Overall, and among the 122 patients analyzed for at least the exon 10 of the *BRCA2* gene, the c.1310_1313delAAGA frameshift mutation was found in 14 patients. Genealogic investigation revealed that all carriers of this mutation shared the same geographic origin and were descendants of the North-East of Morocco.

**Discussion:**

In this study, we highlighted that c.1310_1313delAAGA mutation of *BRCA2* gene is recurrent with high frequency in patients from the North-East region of Morocco. Therefore, we propose to use, in public health strategies, the detection of this mutation as the first-line screening tests in patients with breast and ovarian cancer originated from this region.

## Background

Breast cancer is currently the most common type of cancer in females [1]. The majority are sporadic, where as 5–10% are due to an inherited predisposition to breast and ovarian cancers, transmitted as an autosomal dominant form with incomplete penetrance [2, 3]. Germline mutations of *BRCA1* and *BRCA2* genes are involved in nearly 10% of ovarian cancers and 3–5% of breast cancers respectively [4–6]. Various selection criteria, based on family history, age at onset and tumors clinico-pathological features, as well as computational risk prediction models are used to offer a *BRCA*1/2 testing to patients. *BRCA1* and *BRCA2* are large tumor suppressor genes containing 5592 and 11,385 nucleotides spread over approximately 100,000 bases of genomic DNA each [7]. More than 1833 *BRCA1* and 1552 *BRCA2* mutations distributed throughout the coding regions and flanking intronic sequences, are reported in the Human Gene Mutation Database at the Institute of Medical Genetics in Cardiff (HGMD, http://www.hgmd.cf.ac.uk/ac/index.php). The identification of *BRCA1/2* mutation has a beneficial impact on the management and access to specific treatments (i.e., PARP inhibitors) for patients, and for counseling of relatives at risk, in order to reduce breast cancer mortality [8]. In that sense, the screening of *BRCA1/2* mutations has long moved from the research lab to the clinic as a routine clinical genetic testing by conventional sanger sequencing and more recently by next generation sequencing. However, these technologies remain too expensive and cannot be routinely applied in less privileged countries. To bypass this restriction, target screening of ethnic founder mutations, when possible, may be a useful alternative in public health strategies of developing countries.

The mutational spectrum of *BRCA1/2* in Moroccan population is becoming partially characterized, thanks to few local genetic centers, including our own, which developed oncogenetics consultation for familial forms of breast and ovarian cancers and molecular analysis of *BRCA* genes. However, the large size of *BRCA* genes, the broad spectrum of mutations and the still high cost of genetic testing restrict access of all Moroccan families to oncogenetics services. Thus, identification of a homogenous mutational ethnic background may facilitate the implementation of an effective cost targeted approach for genetic testing in Morocco.

Until shortly, the c.798_799delTT mutation of *BRCA1* was identified several time and noticed as the most recurrent in Moroccan patients from different regions of the country [9–11] (Personal data). We recently reported a further *BRCA2* mutation (c.1310_1313delAAGA; p.Lys437IlefsX22) in three unrelated patients, all from the North-East of Morocco [12]. We aimed in the present study to evaluate the frequency and geographic distribution of this *BRCA2* frameshift, in order to access a first-line *BRCA* genetic testing strategy for Moroccans.

## Patients and methods

### Patients and families

We enrolled 74 female patients with suggestive inherited predisposition to breast and ovarian cancers. They were originated from different regions of Morocco, referred to oncogenetics consultation of our center by the National Institute of Oncology in Rabat, between 2005 and 2015 periods. Second, two groups of 39 and 9 patients from hospitalo-universitary center of Fes and Oujda cities respectively were also investigated. All patients had breast cancer diagnosed at age 50 or younger and/or positive family history with more less one of these criterias: bilateral breast cancer, relative male with breast cancer, triple-negative (estrogen receptor negative, progesterone receptor negative, and HER2/neu [human epidermal growth factor receptor 2] negative) breast cancer, ovary or pancreas or prostate cancer in the same individual or on the same side of the family. Genealogic investigation was detailed for all the patients for at least three generations. All subjects gave written informed consent to the study, which was performed in accordance with the Declaration of Helsinki protocols and approved by the local institutional review boards.

Genomic DNAs were extracted from whole blood samples collected on EDTA using varied protocols, mostly the salting-out method [13], or the QIAamp DNA Blood Mini Kit (Qiagen, Inc.). The quality and quantity of the DNA were controlled by A260/A280 using a Nanodrop spectrophotometer (Fisher Scientific, Wilmington, DE) or Qubit dsDNA HS (High Sensitivity) Assay Kit with Qubit^®^ Fluorometer (Thermo Scientific™, Invitrogen™) according to the manufacturer’s instructions. Aliquots (50**–**100 mL) of packed blood cells were stored at 4 °C until analysis.

According to available resources of our lab and enrolled families, 51 patients were analysed by the conventional individual exon-by-exon Sanger sequencing, whereas only 23 patients were able to benefit from *BRCA* Next generation sequencing as previously reported [12] (Personal data). Second, we performed a target screening of the c.1310_1313delAAGA mutation at exon 10 of *BRCA2* gene in DNA patients monitored at Fes and Oujda centers.

Primers used for PCR amplification of exon 10 of *BRCA2* gene DNA fragments and Sanger sequencing were as follows: BRCA210.2F 5′-TGGAACCAAATGATACTGATCC-3′; BRCA210.2R 5′-TTTCCAGTCCACTTTCAGAGG-3′.

The PCR conditions were carried out in a volume of 50 µL. The mix was submitted to an ABI 9700 thermal cycler (Applied Biosystems) for the gene amplification. PCR protocol included an initial denaturation of 95 °C for 1 min, followed by 35 cycles of 95 °C for 15 s, 59 °C for 15 s, and 72 °C for 10 s.

All used primers were designed by the authors using UCSC https://genome.ucsc.edu/ and checked by SNPCheck3 https://secure.ngrl.org.uk/SNPCheck/snpcheck.htm and In Sillico PCR https://genome.ucsc.edu/cgi-bin/hgPcr (available upon request). Then, amplified fragments were purified and sequenced on an automated ABI prism 3130 DNA sequencer (life technologies) using Big Dye Terminator Kit (life technologies, Foster city, CA). Obtained sequences were aligned to the *BRCA1* and *BRCA2* reference genomic sequence (GenBank: NM_007294 and NM_000059 respectively). The mutation nomenclature was designated according to the Human Genome Variation Society (http://www.hgvs.org).

## Results

The recurrent mutation c.1310_1313delAAGA mutation of *BRCA2* gene, was found in 10 of the 74 patients originated from different regions of Morocco referred to oncogenetics consultation of our center by the National Institute of Oncology in Rabat. From the 39 patients originated from different regions of Morocco referred to hospitalo-universitary center of Fes, 2 patients had the recurrent mutation. 2 of the 9 patients originated from the North East region, have this mutation.

**Fig. 1:**
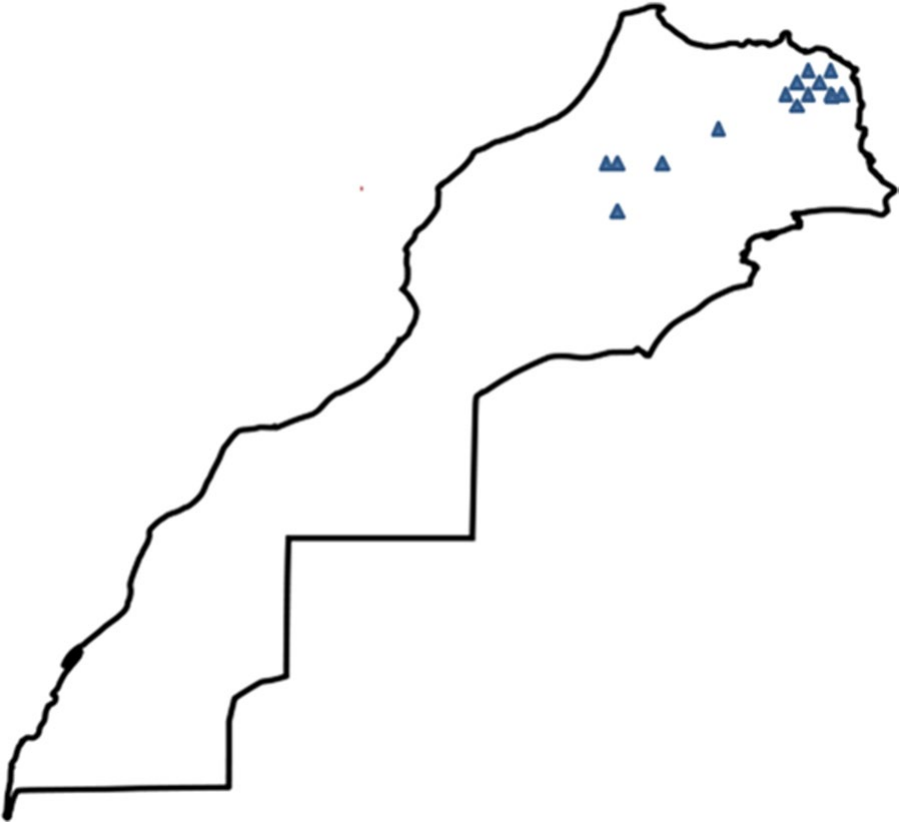
Map of Morocco showing the geographic origin of the 14 patients carrying the c.1310_1313delAAGA mutation. It reveals the concentration of patients in the North-East of Morocco

In total, the c.1310_1313delAAGA frameshift was found in 14 patients among 123 screened for this pathogenic variant. Genealogic investigation showed that all carriers shared the same geographic origin in the North-East of Morocco (Fig. 1).

The c.1310_1313delAAGA; p.Lys437IlefsX22 mutation is already listed and reported deleterious in the Breast Cancer Information Core (BIC) database (http://www.research.nhgri.nih.gov/bic/), the HGMD (http://www.hgmd.cf.ac.uk/ac/index.php), Clinvar (http://www.ncbi.nlm.nih.gov/clinvar/) and previous publications.

## Discussion

Morocco has set up since 2005 a national program against breast cancer, in order to prevent, early detect and properly treat patients. Germline mutations of *BRCA/2* are involved in nearly 10% of ovarian cancers and 3–5% of breast cancers respectively [4–6]. But, despite their rarity, an appropriate strategy of management and prevention of individuals of such families is required because of the high risk of cancers. To date, only a limited number of studies on *BRCA* genetic screening in Moroccan patients is available [9, 10, 14, 15]. Therefore, the prevalence and spectrum of *BRCA1/2* mutations in our population remains mostly unknown. For over a decade, our center provides oncogenetics services and offer genetic testing for certain inherited forms of cancer with special focus on breast cancer. Even more, we conducted presymptomatic diagnosis for a preventive management in some healthy Moroccan females with a high risk of developing breast cancer [14]. However, the expensive cost of *BRCA* screening with the limited funding and the absence of a targeted molecular testing strategy restrict access to these services at all Moroccan patients.

As stated in certain countries and ethnic communities where the *BRCA1/2* mutation screening is limited to a few founder mutations (i.e., c.68_69delAG (BIC: 185delAG) for Ashkenazi Jewish individuals), it will be useful to characterize *BRCA* mutations landscape in Moroccans, in order to set up a specific cost-efficient strategy for screening of recurrent/founder mutations [16]. In this meaning, we evaluated the frequency of the c.1310_1313delAAGA, as we recently noticed in our cohort that it may be the most presumable recurrent mutation [12] (Personal data). Interestingly, 14 patients were carried of the same c.1310_1313delAAGA frameshift. Even more striking, all patients with this recurrent pathogenic variant are originated from a restricted geographic area of the country, suggesting a founder effect of this mutation in the North-East of Morocco (Fig. 1). Furthermore, the *BRCA2* frameshift was not identified in any of the other patients originated from the rest of the country. We assume that there is no selection bias, as our center had a national activity and thus patients originated from different areas of Morocco. According to the Breast Cancer Information Core database (BIC; http://research.nhgri.nih.gov/bic/), this mutation was found in different European patients from Italy, Danemark, Holland and Belgium. It was also recorded 30 times in the French UMD-*BRCA2* database and classified as founder mutation [17]. The pooled genetic background of Moroccans and Europeans suggests a common founder ancestor for this mutation between the two populations. In neighboring countries, c.1310_1313delAAGA was the most recurrent as found in four subjects among 66 Tunisian patients [18]. In a Tunisian review on founder mutations in this population, the hereditary breast and ovarian cancer syndrome was considered as a disease with both Tunisian specific and shared founder mutations, including the c.1310_1313delAAGA mutation of *BRCA2* [19]. In a total of 70 Algerian families, the c.1310_1313delAAGA mutation was found in one family among 10 with *BRCA* mutation [20]. This common geographical distribution of the *BRCA2* frameshift to close maghrebian countries (Algeria, Morocco and Tunisia) could be explained by geographical proximity and migration flow history. Much further from North-African area, the c.1310_1313delAAGA mutation, was carried by one Korean subject among the 31 patients with *BRCA* mutations of the 134 unrelated Korean hereditary breast cancer patients tested [21]. The c.1310_1313delAAGA presumably occurred several times at unstable mutational hot spots parts of the gene in different ethnic groups. Other mutations of BRCA genes were found in Moroccans, and are reported in the Moroccan National Genetic Database (http://ethnos.findbase.org/home-ma).

In conclusion, we reported here that c.1310_1313delAAGA mutation of *BRCA2* gene is at high frequency in the North-East region of Morocco. Therefore, we propose, in the light of this study, to screen firstly, in public health strategies, for the c.1310_1313delAAGA mutation in patients originated from this region.
